# Association between vitamin D status and cardiometabolic risk factors in adults with type 2 diabetes in Shenzhen, China

**DOI:** 10.3389/fendo.2024.1346605

**Published:** 2024-02-14

**Authors:** Yan-Jing Liu, Jing-Wen Duan, Dong-Hui Lu, Fan Zhang, Hong-Li Liu

**Affiliations:** ^1^ Department of Endocrinology and Metabolism, Peking University Shenzhen Hospital, Peking University Shenzhen Hospital, Shenzhen, Guangdong, China; ^2^ Department of Cardiology, The Third Xiangya Hospital, Central South University, Changsha, China; ^3^ Clinical Research Center, The Third Xiangya Hospital, Central South University, Changsha, China

**Keywords:** vitamin D, blood pressure, HbA1c, C-peptide, lipid profiles

## Abstract

**Background:**

Evidence of vitamin D status and cardiometabolic health in adults with type 2 diabetes mellitus (T2DM) is still limited. This study aimed to investigate the association between vitamin D status and cardiometabolic risk factors among adults with T2DM in Shenzhen, China.

**Methods:**

This cross-sectional study included 164 adults (aged ≥18 years) with T2DM who were hospitalized at Peking University Shenzhen Hospital from March 1, 2023, to May 31, 2023. Serum 25-hydroxyvitamin D [25(OH)D] concentration, the active marker of vitamin D, and three major cardiometabolic risk factors including blood pressure (BP), glucose metabolism-related indicators, and blood lipid profiles were collected. Vitamin D deficiency (VDD) was defined as 25(OH)D < 20 ng/mL. Correlation, Regression, and Logistic analysis were applied to verify the association among serum 25(OH)D concentration, VDD, and 11 cardiometabolic risk factors.

**Results:**

Median 25(OH)D concentration was 21.78 [interquartile range (IQR)=17.51-28.05] ng/mL. The prevalence of VDD was 40.24%. Serum 25(OH)D concentration was significantly negatively correlated with diastolic BP (DBP) and glycated hemoglobin A1c (HbA1c) rather than systolic BP, plasma glucose, plasma C-peptide, and blood lipid profiles among adults with T2DM in both correlation and linear regression analysis. Furthermore, the adjusted odd ratio for poor DBP control (≥90 mmHg) of T2DM patients with VDD was 3.164 (95% confidence interval=1.303, 7.683; *P*=0.011) compared to those without VDD.

**Conclusion:**

In China, VDD was highly prevalent among adults with T2DM and associated with greater cardiovascular risk factors, especially with increased chances of uncontrolled DBP. These findings suggest that vitamin D levels should be monitored in T2DM patients, especially those with high DBP.

## 1 Introduction

Diabetes mellitus (DM), especially type 2 DM (T2DM), is known as one of the most prevalent chronic, life-threatening diseases worldwide. According to the latest estimates from the International Diabetes Federation, there are 536.6 million adult (20-79 years old) people with DM in 2021, which represents more than 10% of the global adult population (1). Compared to people without T2DM, in addition, those with T2DM are at higher risk of cardiovascular disease (CVD), which is a well-known cause of morbidity and mortality (2). Although advanced progress has been made in preventing future CVD risk among patients with T2DM, mainly due to early screening and treatment of its risk factors like blood pressure (BP), blood lipid, and glucose metabolism, CVD remains the main cause of death among patients with T2DM (3). Thus, the identification of other modifiable risk factors is of great significance in postponing the development of CVD among patients with T2DM.

Over the last decade, along with the discovery of the expression of vitamin D receptors in non-skeletal organs and tissues, including adipose, cardiomyocytes, pancreatic β-cells, and others, vitamin D status has been described to be linked with the onset of T2DM and the development of complications among patients with T2DM, including CVD (4–8). Also, increasing studies have linked vitamin D status with the common and important cardiometabolic risk factors including BP, blood lipids profile, and glucose metabolism-related indicators in the non-diabetes population. However, the association between vitamin D and these cardiometabolic risk factors remains controversial and limited, especially for patients with T2DM. To date, only two studies have investigated the association between vitamin D status and BP and hypertension, respectively, and one of these studies simultaneously looked at its association with dyslipidemia among patients with T2DM according to the recent systematic review by Md et al. (9–11) In terms of glucose metabolism, most results of related studies are consistent, suggesting that lower concentrations of serum vitamin D are closely related to poor glycemic control, however, these studies were limited to the general population, the older, pregnant women, and other nondiabetic populations, and few studies focused on patients with T2DM, who are more prone to vitamin D deficiency (VDD) (12–18).

Above all, the association between vitamin D status and BP, blood lipids, and glucose metabolism remains further explored. Thus, this study aims to comprehensively explore the association of serum 25(OH) level, the most common active marker of vitamin D, and VDD with BP [including systolic BP (SBP) and diastolic BP (DBP)], glucose metabolism-related indicators [including oral glucose tolerance test (OGTT) that includes both fasting plasma glucose (FPG) and 2h post-load plasma glucose (2hPG), fasting plasma C-peptide, glycated hemoglobin A1c (HbA1c), and insulin resistance (IR)], and blood lipids profile [including total cholesterol (TC), high-density lipoprotein-cholesterol (HDL-c), low-density lipoprotein-cholesterol (LDL-c), and triglyceride (TG)] among adult T2DM patients in China, to provide more theoretical clues for reducing risk of CVD in this population.

## 2 Materials and methods

### 2.1 Study population and design

In this cross-sectional study, we initially included 488 adults (aged ≥18 years) with DM who were hospitalized in the endocrinology units of the Peking University Shenzhen Hospital from March 1, 2023, to May 31, 2023. First of all, those well-diagnosed with DM were included according to the Chinese Guidelines for the Prevention and Treatment of Type 2 Diabetes (2020 edition) as follows (19): 1) FPG  ≥ 7.0 mmol/L, or 2h post glucose load BG ≥ 11.1 mmol/L, or HbA1c  ≥ 6.5%; 2) self-reported diagnosis of DM in a secondary or tertiary hospital, or 3) currently receiving hypoglycemic therapy, including oral hypoglycemic agents and the use of insulin. Then, we excluded those with a diagnosis of unclassified DM, type 1 DM, gestational DM, and other special types of DM. Next, we further excluded participants with conditions that could affect the production and secretion of 25(OH)D, which included hyperparathyroidism, hypoparathyroidism, and hepatic insufficiency.

**Figure 1:**
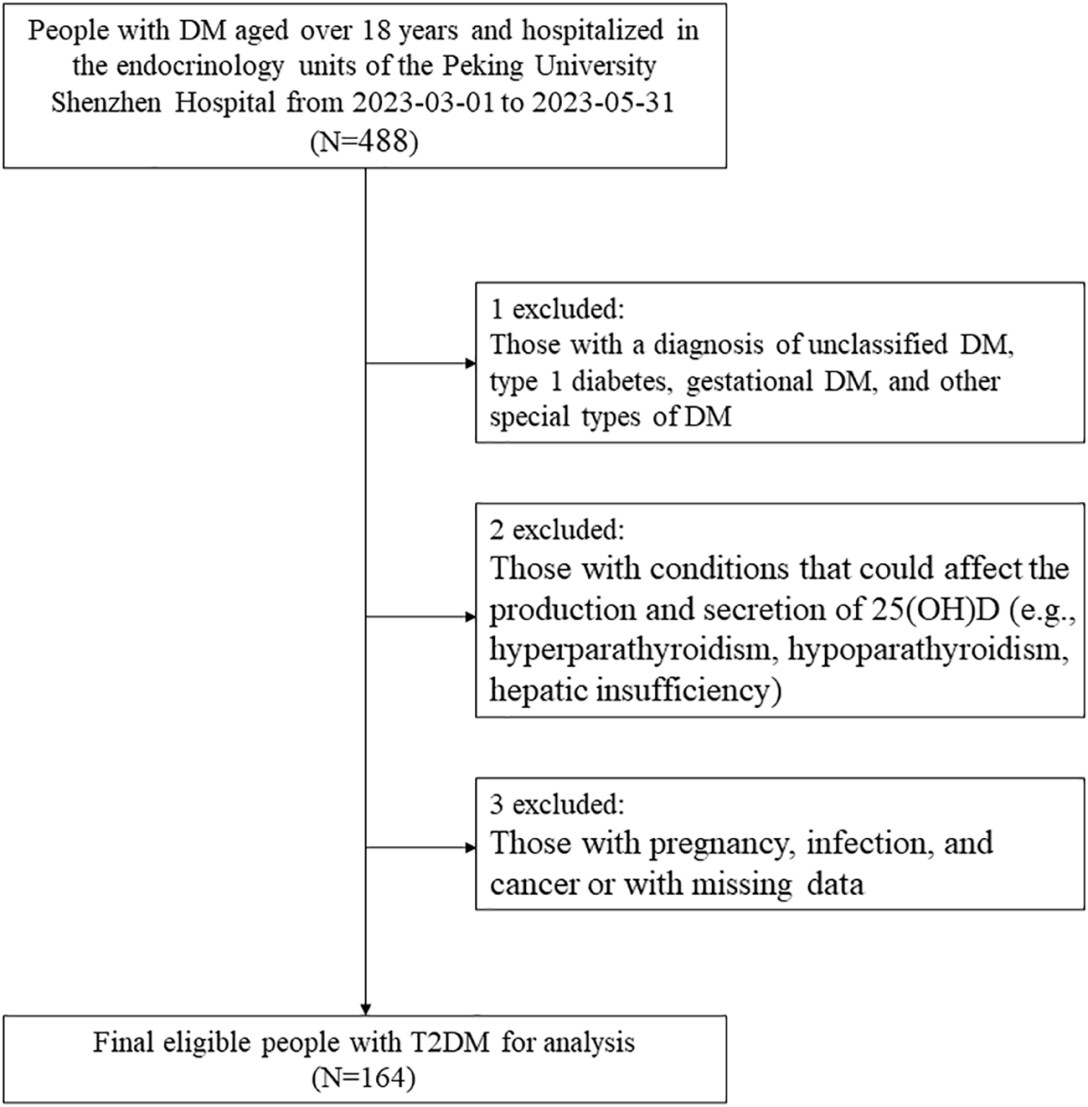
Study flowchart. DM, diabetes; 25(OH)D, 25-hydroxyvitamin D; T2DM, type 2 diabetes mellitus.

In addition, participants with pregnancy, infection, and cancer or missing data were also excluded. Finally, 164 patients with diagnosed T2DM were included in the analysis. The flowchart of the study population is shown in **Figure 1**. This study followed the guidelines of the Declaration of Helsinki and was approved by the Ethics Committee of the Peking University Shenzhen Hospital. All the participants signed an informed consent form at admission and agreed to share their health information for medical research.

### 2.2 Data collection

The basic information, clinical assessment, and biochemical indicators (serum) of all participants were collected from the Hospital Information System (HIS), including age, sex (female/male), weight, height, SBP, DBP, 25(OH)D, TC, TG, LDL-c, HDL-c, HbA1c, FPG, 2hPG, fasting plasma C-peptide, calcium (Ca), calcitonin, parathyroid hormone (PTH), osteocalcin (OC), β-C-terminal cross-linked telopeptide of type I collagen (β-CTX), and procollagen-1 N-terminal peptide (P1NP), creatinine (Cr), calcitonin (CT), uric acid (UA), urinary Albumin-to-Creatinine Ratio (uACR), 24-hour urinary protein quantity (UP), alanine aminotransferase (ALT), thyrotropin (TRH), thyroid stimulating hormone (TSH), and free thyroxine (FT4).

### 2.3 Clinical assessment

Each participant’s weight, height, and BP were measured by well-trained nurses at the time of admission. Specifically, weight and height were measured with the participants wearing light clothing and no shoes to the nearest 0.1 kg and 0.1 cm, respectively. Body mass index (BMI) was calculated as weight in kilograms (kg) divided by the square of height in meters (m). SBP and DBP of the right upper arm were measured using a validated digital automatic analyzer (Omron, Japan) in a seated position after at least 5 minutes of rest and an additional recording after a 2-minute break. SBP and DBP were each measured twice and the mean of the two readings was used for analyses. If the two readings differed by >5 mmHg, a third measurement was performed, and the average of all three readings was applied.

### 2.4 Biochemical measurements

Blood samples of each participant who was included in the final analysis were drawn in the morning after overnight fasting (≥8 hours) during hospitalization. The main outcome parameters of the laboratory included blood lipids (TC, TG, LDL-c, HDL-c) and glucose metabolism-related indicators (HbA1c, FPG, 2hPG, and fasting plasma C-peptide). Participants underwent a 75 g OGTT with plasma glucose measured using a routine hexokinase method and C-peptide measured by Chemiluminescence immunoassay before (fasting) and 2 h post glucose load (i.e., FPG, 2hPG, and fasting plasma C-peptide). HbA1c was measured using capillary electrophoresis. TG, TC, and LDL-c/HDL-c were measured using enzymatic assays (GK-GPO-POD colorimetric method), cholesterol oxidase methods, and homogeneous methods, respectively. Levels of all the above parameters were measured by an automatic biochemical analyzer (AU5800 Series Chemistry Analyzers, Beckman Coulter) in the clinical laboratory of Peking University Shenzhen Hospital.

### 2.5 Measurement of serum 25(OH)D and classification of vitamin D status

Serum 25(OH)D concentration was measured by electrochemiluminescence immunoassay (ECLIA) using the same automatic biochemical analyzer in the clinical laboratory of Peking University Shenzhen Hospital. VDD was defined as 25(OH)D <20 ng/mL, and vitamin D sufficiency (VDS) was defined as 25(OH)D ≥20 ng/mL according to the Endocrine Society clinical practice guidelines and methods provided by serval published research (20).

### 2.6 Definition of outcomes

The cardiometabolic risk factors of interest in this study include BP (including SBP and DBP), glucose metabolism-related indicators (including FPG, 2hPG, fasting plasma C-peptide, HbA1c, and IR), and blood lipid profile (including TC, TG, LDL-c, and HDL-c). Usually, IR is expressed by HOMA, which can calculated from fasting blood insulin, blood glucose, and C-peptide values. Since most patients with T2DM may use insulin to control blood glucose levels, the measurement of fasting insulin may be affected by exogenous insulin; therefore, C-peptide values were used to calculate HOMA in this study [i.e., HOMA2 based on C-peptide (HOMA2-Cpep)]. According to the method provided by Ferrannini G et al. (21), HOMA2-Cpep was calculated at https://www.dtu.ox.ac.uk/homacalculator/, with high HOMA-IR values indicating insulin resistance.

Poor BP control included the following two situations: SBP ≥140 mmHg or DBP ≥90 mmHg. Poor blood lipid control was defined as the following four situations: hypertriglyceridemia (TG ≥2.26 mmol/L), hypercholesterolemia (TC ≥6.2 mmol/L), high levels of LDL-c (LDL-c ≥4.14 mmol/L), or low levels of HDL-c (HDL-c ≤1.04 mmol/L). Poor glycemic control was defined as the occurrence of any one of the following three situations: HbA1c ≥7%, FPG ≥ 7.0 mmol/L, or 2h post glucose load BG ≥ 11.1 mmol/L.

### 2.7 Statistical analysis

Baseline characteristics of the participants classified by vitamin D status were presented as means ± standard deviation (SD) or median [interquartile range (IQR)] for continuous variables with normal or skewed distribution, respectively; and percentages (%) for categorical variables. To compare the differences between VDD and VDS groups, the student T-test or Mann-Whitney U test was performed for continuous variables with normal or skewed distribution, respectively; and the Chi-square test was performed for categorical variables. Pearson correlation analysis or Spearman correlation analysis for continuous variables with normal or skewed distribution were used to analyze whether 25(OH)D concentrations linked to SBP, DBP, HbA1c, FPG, 2hPG, fasting plasma C-peptide, HOMA2-Cpep, TC, TG, LDL-_C_, and HDL-_C_, respectively.

To determine whether 25(OH)D levels independently affected these indicators, multivariable linear regression analysis was further performed by three models (model 1: unadjusted; model 2: adjusted for age and sex; and model 3: adjusted for age, sex, BMI, ALT, CR, TSH, and FT4). Furthermore, multivariable logistic analysis was used to clarify the association between VDD and these risk factors that were significant in the correlation analysis and linear regression analysis based on the same models. All statistical analysis was performed by Stata statistical software version 17.0 (Stata Corp LP, College Station, TX, United States). The value of P < 0.05 was considered statistically significant.

## 3 Results

### 3.1 Characteristics of the study population

A total of 164 adults with T2DM with a mean age of 58.2 ± 11.0 years met the criteria and were finally enrolled in this study. The characteristics of these participants are shown in **Table 1**. The median 25(OH)D concentration in the present study was 21.78 (IQR=17.51-28.05) ng/mL. Overall, 66 (40.24%) participants were confirmed to have VDD. In addition, adults with T2DM in the VDD group exhibited significantly elevated DBP (80.91 ± 11.04 vs 74.76 ± 10.67; *P=*0.001), β-CTX [0.45 (0.31-0.58) vs 0.36 (0.23-0.51); 0.048], HbA1c (8.69 ± 1.70 vs 8.03 ± 1.69; 0.016), and uACR [21.66 (7.40-67.60) vs 10.89 (5.68-27.61); 0.038], whereas those in the VDS group exhibited significantly higher age (55.7 ± 11.6 vs 59.9 ± 10.3; 0.014).

**Table 1:** Baseline characteristics of the study participants.

Characteristics	All participants	Vitamin D deficiency (<20 ng/mL)		Vitamin D sufficiency (≥20 ng/mL)		*P*-value
		n	Mean ± SD or Median (IQR) ^*^	n	Mean ± SD or Median (IQR)	
Female/male	70/94	28/38	/	42/56	/	0.956
Age, year	58.2 ± 11.0	66	55.7 ± 11.6	98	59.9 ± 10.3	**0.014**
Clinical assessment						
BMI, kg/m^2^	24.06 (22.13-25.90)	66	24.24 (26.30-22.49)	98	23.94 (21.87-25.85)	0.474
SBP, mmHg	122 (112.5-136)	66	122 (113-136)	98	119.5 (108-136)	0.508
DBP, mmHg	77.23 ± 11.20	66	80.91 ± 11.04	98	74.76 ± 10.67	**0.001**
Laboratory assessment (serum)						
Osteocalcin, ng/mL	13.2 (10.45-16.8)	66	14.00 (10.60-17.70)	98	12.5 (10.4-15.9)	0.148
β-CTX, ng/mL	0.395 (0.26-0.54)	66	0.45 (0.31-0.58)	98	0.36 (0.23-0.51)	**0.048**
P1NP, g/mL	35.6 (27.5-48.2)	66	39.35 (29.5-50.5)	98	34 (26.3-46.3)	0.066
25(OH)D, g/mL	21.78 (17.51-28.05)	66	16.73 (14.57-18.43)	98	27.02 (22.98-32.96)	**<0.001**
Ca, mmol/L	2.26 (2.20-2.31)	66	2.27 (2.20-2.31)	98	2.25 (2.19-2.31)	0.365
Cr, μmol/L	67.00 (53.50-78.50)	66	64.00 (51.00-80.00)	98	67.00 (54.00-77.00)	0.735
uACR, mg/g	14.11 (5.75-39.61)	66	21.66 (7.40-67.60)	98	10.89 (5.68-27.61)	**0.038**
24hUP, g/24h	0.11 (0.08-0.16)	66	0.10 (0.06-0.16)	98	0.11 (0.08-0.15)	0.385
PTH, pmol/L	3.4 (2.7-4.5)	34	3.5 (2.9-4.9)	61	3.4 (2.6-4.2)	0.125
Calcitonin, pg/ml	0.58 (0.50-2.02)	32	0.50 (0.50-1.34)	54	0.77 (0.50-2.14)	0.211
UA, μmol/L	330.18 ± 85.50	66	336.36 ± 84.95	98	326.01 ± 86.05	0.449
ALT, U/L	19.34 ± 8.55	66	20.55 ± 9.10	98	18.52 ± 8.09	0.291
TSH, mIU/L	1.71 (1.09-2.38)	64	1.77 (1.14-2.60)	98	1.60 (0.97-2.14)	0.231
FT4, pmol/L	11.18 (9.95-12.74)	66	11.25 (10.01-13.00)	98	11.12 (9.75-12.61)	0.837
Glucometabolic status						
FPG, mmol/L	6.66 (5.68-8.50)	66	7.41 (6.02-9.04)	98	6.46 (5.45-8.09)	0.057
2hPG, mmol/L	16.63 ± 4.84	66	17.01 ± 5.02	98	16.38 ± 4.73	0.418
HbA1c (%)	8.29 ± 1.72	66	8.69 ± 1.70	98	8.03 ± 1.69	**0.016**
Fasting plasma C-peptide, nmol/L	0.44 (0.25-0.67)	66	0.40 (0.27-0.67)	98	0.46 (0.24-0.66)	0.960
HOMA2-Cpep ^#^	1.23 (0.89-1.73)	58	1.17 (0.82-1.79)	80	1.28 (1.00-1.72)	0.449
Lipid profile						
TG, mmol/L	1.28 (0.94-2.06)	66	1.35 (1.00-2.30)	98	1.27 (0.86-1.78)	0.078
TC, mmol/L	4.32 (3.43-5.23)	66	4.34 (3.44-5.24)	98	4.31 (3.42-5.14)	0.667
HDL-c, mmol/L	1.08 (0.93-1.28)	66	1.06 (0.91-1.25)	98	1.09 (0.93-1.34)	0.282
LDL-c, mmol/L	2.73 (2.05-3.38)	66	2.73 (2.16-3.41)	98	2.73 (2.01-3.31)	0.473

^*^Data present as Mean ± SD or Median (IQR) for continuous variables and percentages (%) for categorical variables.

^#^HOMA2-Cpep was calculated at https://www.dtu.ox.ac.uk/homacalculator/.

BMI, body mass index; SBP, systolic blood pressure; DBP, diastolic blood pressure; β-CTX, β-C-terminal cross-linked telopeptide of type I collagen; P1NP, procollagen-1 N-terminal peptide; 25(OH)D, 25-hydroxyvitamin D; Ca, calcium; Cr, creatinine; HbA1c, glycated hemoglobin A1c; uACR, urinary Albumin-to-Creatinine Ratio; UP, 24-hour urinary protein quantity; PTH, parathyroid hormone; TG, triglyceride; TC, total cholesterol; HDL-c, high-density lipoprotein-cholesterol; LDL-c, low-density lipoprotein-cholesterol; ALT, alanine aminotransferase; TSH, thyroid stimulating hormone; FT4, free thyroxine; FBG, fasting plasma glucose; 2hPG, 2h post-load plasma glucose; HOMA2-Cpep, HOMA2 based on C-peptide.

The bold values mean their value less than 0.05, which is statistically significant (i.e., P<0.05).

### 3.2 Serum 25(OH)D concentration and cardiometabolic health

**Figure 2:**
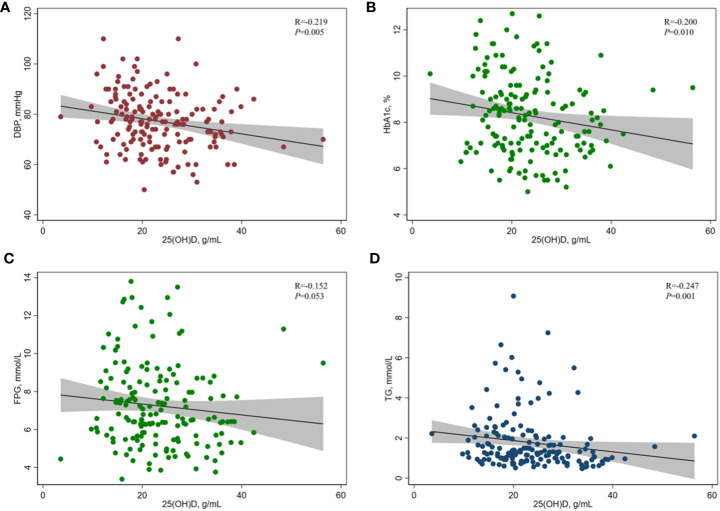
Correlation between serum 25(OH)D concentration and **(A)** DBP, **(B)** HbA1c, **(C)** FPG, and **(D)** TG in adults with T2DM. Spearman rank correlation analysis was used due to the skewed distribution of serum 25(OH)D in this study. 25(OH)D, 25-hydroxyvitamin D; HbA1c, glycated hemoglobin A1c; FPG, fasting plasma glucose; TG, triglyceride; DBP, diastolic blood pressure.

**Table 2** and **Figure 2** show the results of the correlation analysis of 25(OH)D and cardiometabolic risk factors. For both measures of BP, the correlation analysis showed that serum 25(OH)D concentration was only significantly negatively correlated with DBP (r=-0.219, *P=*0.005), but SBP (r=-0.008, *P=*0.922) among adult T2DM patients in China. For different indicators of lipid profile (TC, TG, LDL-_C_, HDL-_C_) and glucose metabolism (HbA1c, FPG, 2hPG, fasting plasma C-peptide, HOMA2-Cpep), serum 25(OH)D concentration was only significantly negatively correlated with TG (r=-0.247, *P=*0.001), FPG (r=-0.152, *P=*0.053), and HbA1c (r=-0.200, *P=*0.010) among this population.

**Table 2:** Correlation between serum 25(OH)D concentrations and cardiometabolic risk factors in adult T2DM patients.

Cardiometabolic risk factors	Serum 25(OH)D concentrations	
	r ^*^	*P*-value
Blood pressure		
SBP, mmHg	-0.008	0.922
DBP, mmHg	-0.242	**0.002**
Glucometabolic status		
HbA1c, %	-0.200	**0.010**
FPG, mmol/L	-0.152	0.053
2hPG, mmol/L	-0.026	0.740
Fasting plasma C-peptide, nmol/L	-0.041	0.604
HOMA2-Cpep	-0.018	0.834
Lipid profile		
TG, mmol/L	-0.247	**0.001**
TC, mmol/L	-0.078	0.324
HDL-c, mmol/L	0.139	0.077
LDL-c, mmol/L	-0.101	0.197

^*^Data are Spearman Correlation Coefficient. Spearman rank correlation analysis was used due to the skewed distribution of serum 25(OH)D in this study.

25(OH)D, 25-hydroxyvitamin D; T2DM, type 2 diabetes mellitus; SBP, systolic blood pressure; DBP, diastolic blood pressure; TG, triglyceride; TC, total cholesterol; HDL-c, high-density lipoprotein-cholesterol; LDL-c, low-density lipoprotein-cholesterol; HbA1c, glycated hemoglobin A1c; FBG, fasting plasma glucose; 2hPG, 2h post-load plasma glucose; HOMA2-Cpep, HOMA2 based on C-peptide.

The bold values mean their value less than 0.05, which is statistically significant (i.e., P<0.05).

The results of multiple linear regression analysis of serum 25(OH)D concentrations and cardiometabolic risk factors are shown in **Table 3**. Among adults with T2DM, serum 25(OH)D concentration were significantly negatively correlated with HbA1c [β (95% CI): -0.037 (-0.070, -0.005), *P*=0.026], TG [-0.028 (-0.054, -0.001), 0.040], and DBP [-0.303 (-0.513, -0.093), 0.005], and they were significantly positively correlated with HDL [0.006 (0.001, 0.012), 0.027]. Similar results were obtained when some or all potential confounders were adjusted, that is, serum 25(OH)D concentration was significantly negatively correlated with HbA1c [-0.040 (-0.075, -0.005), 0.024] and DBP [-0.252 (-0.470, -0.034), 0.024] and significantly positively correlated with HDL [0.007 (0.001, 0.012), 0.016], but there was no significant correlation with TG [-0.015 (-0.0413, 0.012),0.280].

**Table 3:** Regression analyses between serum 25(OH)D concentrations and cardiometabolic risk factors in adult T2DM patients.

Outcomes	Model 1 ^*^		Model 2		Model 3	
	β (95% CI)	*P*-value	β (95% CI)	*P*-value	β (95% CI)	*P*-value
**No. participants**	164		164		162	
Blood pressure						
SBP, mmHg	0.008 (-0.351, 0.366)	0.967	-0.097 (-0.460, 0.267)	0.600	-0.067 (-0.439, 0.306)	0.724
DBP, mmHg	-0.303 (-0.513, -0.093)	**0.005**	-0.244 (-0.453, -0.034)	**0.023**	-0.252 (-0.470, -0.034)	**0.024**
Glucometabolic status						
HbA1c, %	-0.037 (-0.070, -0.005)	**0.026**	-0.035 (-0.069, -0.002)	**0.039**	-0.040 (-0.075, -0.005)	**0.024**
FPG, mmol/L	-0.029 (-0.071, 0.013)	0.177	-0.027 (-0.070, 0.016)	0.222	-.0295 (-0.073, 0.014)	0.185
2hPG, mmol/L	0.008 (-0.085, 0.101)	0.869	-0.015 (-0.109, 0.080)	0.760	-.0235 (-0.120, 0.073)	0.632
Fasting plasma C-peptide, nmol/L	-3.257 (-10.398, 3.885)	0.369	-0.747 (-7.919, 6.425)	0.837	1.913 (-5.118, 8.944)	0.592
HOMA2-Cpep	-0.008 (-0.026, 0.011)	0.415	-0.003 (-0.022, 0.015)	0.726	0.001 (-0.017, 0.019)	0.898
Lipid profile						
TG,	-0.028 (-0.054, -0.001)	**0.040**	-0.023(-0.050, 0.004)	0.096	-0.015 (-0.0413, 0.012)	0.280
TC	-0.013 (-0.039, 0.012)	0.305	-0.009 (-0.035, 0.017)	0.502	-0.003 (-0.029, 0.023)	0.832
HDL-c	0.006 (0.001, 0.012)	**0.027**	0.005 (-0.000, 0.011)	0.067	0.007 (0.001, 0.012)	**0.016**
LDL-c	-0.011 (-0.031, 0.009)	0.264	-0.008 (-0.028, 0.013)	0.466	-0.004 (-0.024, 0.017)	0.726

^*^Model 1: unadjusted; Model 2: adjusted for age and sex; and Model 3: adjusted for age, sex, BMI, ALT, CR, TSH, and FT4.

CI, confidence interval; 25(OH)D, 25-hydroxyvitamin D; T2DM, type 2 diabetes mellitus; SBP, systolic blood pressure; DBP, diastolic blood pressure; TG, triglyceride; TC, total cholesterol; HDL-c, high-density lipoprotein-cholesterol; LDL-c, low-density lipoprotein-cholesterol; HbA1c, glycated hemoglobin A1c; FBG, fasting plasma glucose; 2hPG, 2h post-load plasma glucose; HOMA2-Cpep, HOMA2 based on C-peptide; BMI, body mass index; ALT, alanine aminotransferase; Cr, creatinine; TSH, thyroid stimulating hormone; FT4, free thyroxine.

The bold values mean their value less than 0.05, which is statistically significant (i.e., P<0.05).

### 3.3 Vitamin D deficiency and cardiometabolic health

**Figure 3:**
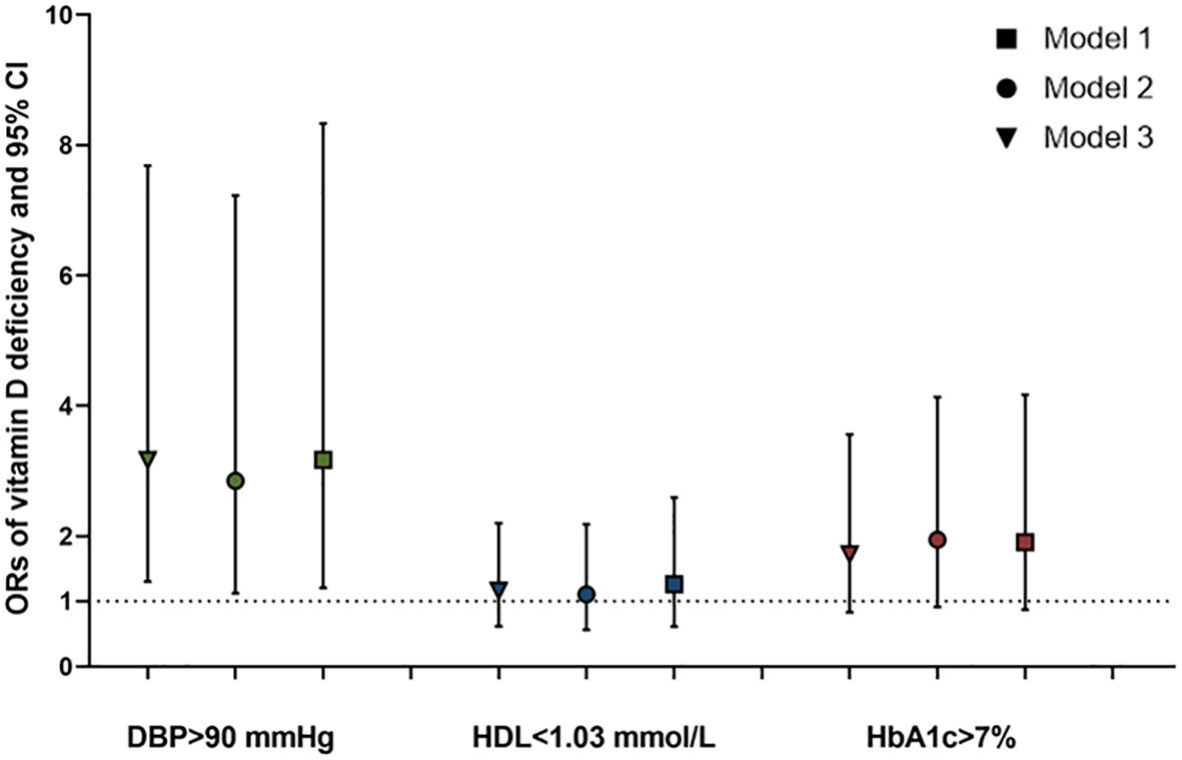
ORs and 95%CI of the vitamin D deficiency for poor control of DBP, HDL, and HbA1c in adults with T2DM. ^*^Model 1: unadjusted; Model 2: adjusted for age and sex; and Model 3: adjusted for age, sex, BMI, ALT, CR, TSH, and FT4. ORs, odds ratios; CI, confidence interval; HbA1c, glycated hemoglobin A1c; DBP, diastolic blood pressure; HDL, high-density lipoprotein-cholesterol; BMI, body mass index; ALT, alanine aminotransferase; Cr, creatinine; TSH, thyroid stimulating hormone; FT4, and free thyroxine.

Compared to adults with T2DM in the VDS group, the logistic analysis showed that the unadjusted odds ratios (ORs) for poor DBP control (≥90 mmHg), low levels of HDL (≤1.04 mmol/L), and poor glycemic control (HbA1c ≥7%) among those in the VDD group were 3.164 (95%CI=1.303, 7.683; P=0.011), 1.163 (0.616, 2.196; 0.640), and 1.800 (0.839, 3.863; 0.131) and adjusted ORs were 3.168 (95%CI=1.303, 7.683; P=0.019), 1.258 (0.611, 2.592; 0.534), and 2.177 (0.942, 5.030; 0.069), respectively (shown in **Figure 3**).

## 4 Discussion

This study demonstrated that there was a significant correlation between serum 25(OH)D concentration and cardiometabolic risk factors of DBP, HbA1c, TG, and HDL-c, especially DBP, in Chinese adults with T2DM. Specifically, lower concentrations of serum 25(OH)D significantly correlated with higher levels of DBP, HbA1c, and TG, but with lower levels of HDL-_C_. These associations were robust after adjusting multiple variables including age, sex, BMI, ALT, Scr, TSH, and FT4. Further analysis revealed that the prevalence of VDD reached 40.24% of the studied population, and adults with T2DM in the VDD group increased the risk of poor DBP control compared to those in the VDS group in China. These findings suggest that adults with T2DM who have poor BP control, especially DBP, should monitor their vitamin D levels and consider taking vitamin D supplementation, if necessary, to reduce future CVD risk.

At present, CVD remains the main cause of death among adults with T2DM (22). For example, a contemporary multi-ethnic population-based observational study reported that the adjusted hazard ratio of CVD events for patients with T2DM was 1.28 (1.09-1.51) in individuals of South Asian ethnicity compared to those without T2DM (23). In addition, poor glycemic control is a major risk factor for the microvascular and macrovascular complications of patients with T2DM, such as retinopathy, diabetic nephropathy, and CVD. In this context, good glycemic control is a priority of T2DM management, but no more than 50% of patients are satisfied with glycemic control, especially in China. Therefore, the identification of risk factors other than the well-known ones such as poor glycemic control, increased BP or hypertension, and obesity is essential to improve glycemic control rates. Over the last decade, the pleiotropic metabolic roles of vitamin D have attracted widespread concern, especially in glucose metabolism and cardiovascular health, along with the discovery of the expression of vitamin D receptors in non-skeletal organs and tissues such as adipose, vascular smooth muscles, cardiomyocytes, and pancreatic β-cells (11). Most of the epidemiologic and meta-analysis studies have reported consistent inverse associations between serum 25(OH)D level and the risk of incident diabetes in the general population and that of poor glycemic control among adults with T2DM. For example, Khan et al. (24) found that vitamin D at baseline is inversely associated with future risks of T2DM in apparently healthy adults. Song et al. (25) combined data from 21 longitudinal cohorts with a total of 76,220 participants and showed that the estimated risk reduction for incident diabetes in the highest versus the lowest category of 25(OH)D was 38%.

In terms of vitamin D and glycemic control of patients with T2DM, several previous research studies have shown that low vitamin D levels have been associated with poor control of FPG and HbA1c. Consistent with these studies, our study found that 25(OH)D levels were significantly inversely associated with HbA1c, but not FBG, in both correlation and linear regression analyses, which provided more evidence that vitamin D may play an important role in the long-term glycemic control of patients with T2DM. The underlying mechanism by which VDD affects glycemic control is not fully understood. In recent years, some studies have suggested that VDD might indirectly decrease the level of intracellular calcium, thereby reducing the level of insulin secretion and beta-cellular dysfunction and, or directly reducing intracellular calcium level, as a result, impaired glucose tolerance (26–28). In addition, vitamin D may reduce systemic inflammation via the vitamin D receptor on pancreatic beta cells and in muscles and the liver to improve peripheral IR. However, our study found no significant association between vitamin D and HOMA2-Cpep, a marker of IR, suggesting that the inverse association of vitamin D with long-term poor glycemic control may not be acting through insulin function and accurate underlying mechanisms need to be further explored in the future.

In addition to glycemic control, poor control of BP and blood lipid are the two main factors contributing to the elevated risk of future CKD in patients with T2DM. For the association between vitamin D and BP, early in the 1980s, Sowers et al. (29) found a significant inverse association between the estimated dietary intake of vitamin D and SBP in younger women. Since then, an increasing number of studies have explored the association between 25(OH)D levels and BP and lipid profile in the general population, overweight/obese population, childhood, and pregnant (30–32). However, few relevant studies have focused on patients with T2DM, although this group has a higher risk of VDD (33). Of them, the case-control study included 80 overweight/obese subjects with T2DM and 77 healthy subjects matched by sex, age, and BMI from Tabriz, identifying that there was a significant negative association between serum 25(OH)D concentrations and DBP among adults with T2DM compared with healthy controls (9). However, another study by Ahmed et al. (10) suggested the association between vitamin D and BP was influenced by vitamin D type, and higher levels of vitamin D2, but not vitamin D3, are associated with hypertension in patients with diabetes. Consistent with the case-control study, this study observed a significant inverse association between 25(OH)D concentrations and DBP, which provides a new possible treatment strategy for T2DM patients with high DBP.

In addition, our study also found that lower 25(OH)D concentrations were associated with poorer lipid management including TG and HDL-_C_, especially lower HDL-c levels, among adults with T2DM. This trend is consistent with some previous studies focusing on the non-diabetes population. The underlying mechanisms by which vitamin D influences the BP and lipid profile have not been fully elucidated. First, it has been recognized that vitamin D might lower BP by suppressing renin synthesis to downregulate the activity of the renin-angiotensin-aldosterone system (RASS) (34). Second, reduced active vitamin D can lead to increased iPTH production in the parathyroid gland and stimulate the expression of PTH2 receptors in vascular smooth muscle cells, thereby up-regulating the expression of the receptor for advanced glycation end products (RAGE) and the production of monocyte-macrophage cytokines and IL-6, as a result, promoting calcium deposition in the arterial wall, leading to collagen deposition, and increased vascular stiffness (35). These pathways can also affect lipid metabolism, for example, high iPTH levels may also accelerate calcium influx into adipocytes, thereby increasing lipase expression, and then increasing various lipid factors (36). Furthermore, vitamin D can inhibit the synthesis and excretion of TG by promoting the intestinal absorption of calcium. Furthermore, vitamin D may participate in the reverse cholesterol transport process by regulating the efflux of cholesterol from cholesterol-carrying macrophages, which removes excess cholesterol from the liver, thus causing an increase in HDL levels (37). But for now, why VDD is only significantly associated with DBP in BP and with HDL in lipid profile needs to be further verified and explored by studies with larger sample sizes.

### 4.1 Strengths and limitations

To the best of our knowledge, this is the first population-based study from China to simultaneously compare the association between vitamin D status and multiple cardiovascular risk factors in the same population with T2DM. Meanwhile, it should be noted that our study also has some limitations. First, this study is a cross-sectional study, the results of which cannot determine the causal association. Second, several vital potential indicators were not included in the final analysis, such as the duration of T2DM and whether use of VD supplements. Moreover, the sample size of this study was relatively small.

## 5 Conclusions

In conclusion, this population-based study showed serum 25(OH)D concentration was significantly associated with multiple cardiometabolic risk factors including DBP, HbA1c, TG, and HDL-c among adults with T2DM, especially with DBP, which suggests that vitamin D may contribute to increased CVD risk through poor management of DBP in patients with T2DM. The role of vitamin D in the management of BP, especially DBP, and this role in the increased risk of CVD among adults with T2DM deserves further investigation.

## Data availability statement

The original contributions presented in the study are included in the article/supplementary material, further inquiries can be directed to the corresponding author/s.

## Ethics statement

The studies involving humans were approved by Medical Ethics Committee of Peking University Shenzhen Hospital. The studies were conducted in accordance with the local legislation and institutional requirements. The participants provided their written informed consent to participate in this study.

## Author contributions

Y-JL: Methodology, Writing – original draft, Writing – review & editing, Data curation, Formal analysis, Investigation. J-WD: Project administration, Writing – review & editing, Conceptualization, Methodology, Validation, Visualization, Writing – original draft. D-HL: Investigation, Writing – review & editing. FZ: Investigation, Writing – review & editing. H-LL: Investigation, Writing – review & editing, Data curation, Project administration.
